# Selective pressure against horizontally acquired prokaryotic genes as a driving force of plastid evolution

**DOI:** 10.1038/srep19036

**Published:** 2016-01-11

**Authors:** Briardo Llorente, Flavio S. J. de Souza, Gabriela Soto, Cristian Meyer, Guillermo D. Alonso, Mirtha M. Flawiá, Fernando Bravo-Almonacid, Nicolás D. Ayub, Manuel Rodríguez-Concepción

**Affiliations:** 1 Centre for Research in Agricultural Genomics (CRAG) CSIC-IRTA-UAB-UB, 08193 Barcelona, Spain; 2 Instituto de Investigaciones en Ingeniería Genética y Biología Molecular Dr. Héctor Torres, Consejo Nacional de Investigaciones Científicas y Técnicas (INGEBI-CONICET), C1428ADN Buenos Aires, Argentina; 3 Departamento de Fisiología, Biología Molecular y Celular, Facultad de Ciencias Exactas y Naturales, Universidad de Buenos Aires, C1428EGA Buenos Aires, Argentina; 4 Departamento de Ciencia y Tecnología. Universidad Nacional de Quilmes, B1876BXD Bernal, Argentina; 5 Instituto de Genética Ewald A. Favret, Centro de Investigación en Ciencias Veterinarias y Agronómicas, Instituto Nacional de Tecnología Agropecuaria (CICVyA-INTA), B1712WAA Castelar, Argentina; 6 Consejo Nacional de Investigaciones Científicas y Técnicas (CONICET), C1033AAJ Buenos Aires, Argentina

## Abstract

The plastid organelle comprises a high proportion of nucleus-encoded proteins that were acquired from different prokaryotic donors via independent horizontal gene transfers following its primary endosymbiotic origin. What forces drove the targeting of these alien proteins to the plastid remains an unresolved evolutionary question. To better understand this process we screened for suitable candidate proteins to recapitulate their prokaryote-to-eukaryote transition. Here we identify the ancient horizontal transfer of a bacterial polyphenol oxidase (PPO) gene to the nuclear genome of an early land plant ancestor and infer the possible mechanism behind the plastidial localization of the encoded enzyme. Arabidopsis plants expressing PPO versions either lacking or harbouring a plastid-targeting signal allowed examining fitness consequences associated with its subcellular localization. Markedly, a deleterious effect on plant growth was highly correlated with PPO activity only when producing the non-targeted enzyme, suggesting that selection favoured the fixation of plastid-targeted protein versions. Our results reveal a possible evolutionary mechanism of how selection against heterologous genes encoding cytosolic proteins contributed in incrementing plastid proteome complexity from non-endosymbiotic gene sources, a process that may also impact mitochondrial evolution.

A large number of plant genes encoding plastid proteins originated via independent horizontal gene transfer (HGT) events from prokaryotes other than *Cyanobacteria*^1^,^2^, the accepted endosymbiotic precursor of modern-day plastids^3^. The vast majority of those genes are nuclear, and therefore their protein products are translated in the cytosol and subsequently imported into the plastid. This in itself is remarkable, because prokaryotic genes lack the information needed to target their encoded proteins to eukaryotic organelles and hence must have acquired it following insertion in the nuclear genome and transcriptional activation. Despite the profound evolutionary importance of this process, we know little about the forces underlying the targeting of these alien proteins to the plastid.

We addressed this conundrum using a novel approach that is based on the idea that studying the ancestral (cytosolic) and modern (plastidial) subcellular localization of HGT-derived proteins *in vivo* may provide clues about the evolutionary mechanisms that ultimately resulted in their organellar targeting. We accordingly screened for candidate proteins of non-photosynthetic function that were present exclusively in prokaryotes and plants, and whose plastidial localization in the later could not be explained by a strict functional requirement. A uniquely suited candidate for a case study was found to be polyphenol oxidase (PPO; Enzyme Commission 1.14.18.1 or 1.10.3.1), a bacterial enzyme^4^ whose nucleus-encoded plant homologues are almost ubiquitously present in plastids^5^. Although the physiological functions of PPO are not fully understood in plants, its capability to oxidize phenolic compounds (e.g. phenylpropanoids) into highly reactive quinones is thought to function as an “armed bomb” defence mechanism against phytophagous organisms functioning only when cell damage occurs and plastids break^6^,^7^,^8^. In bacteria, PPO enzymes are involved in the production of melanin-like polymers that protect bacterial cells and spores against UV radiation, heat, metal ions, oxidants, enzymatic hydrolysis, antimicrobial compounds and phagocytosis^9^.

We present here evidence indicating that an early common ancestor of land plants acquired a bacterial gene coding for PPO through an ancient HGT event during the plant colonization of land. Recapitulating the bacteria-to-plants transition of PPO in the model plant *Arabidopsis thaliana* (Brassicaceae; hereafter Arabidopsis) suggests that the plastidial targeting of PPO in plants is likely a consequence of selective pressures acting against the deleterious effects caused by a PPO enzyme functioning in the cytosol. Given that biological processes in eukaryotic cells are highly compartmentalized, selection against horizontally acquired genes encoding non-targeted heterologous proteins may represent a general route by which novel organellar protein versions can arise.

## Results

### Plants horizontally acquired a bacterial *PPO* gene during colonization of land

**Figure 1:**
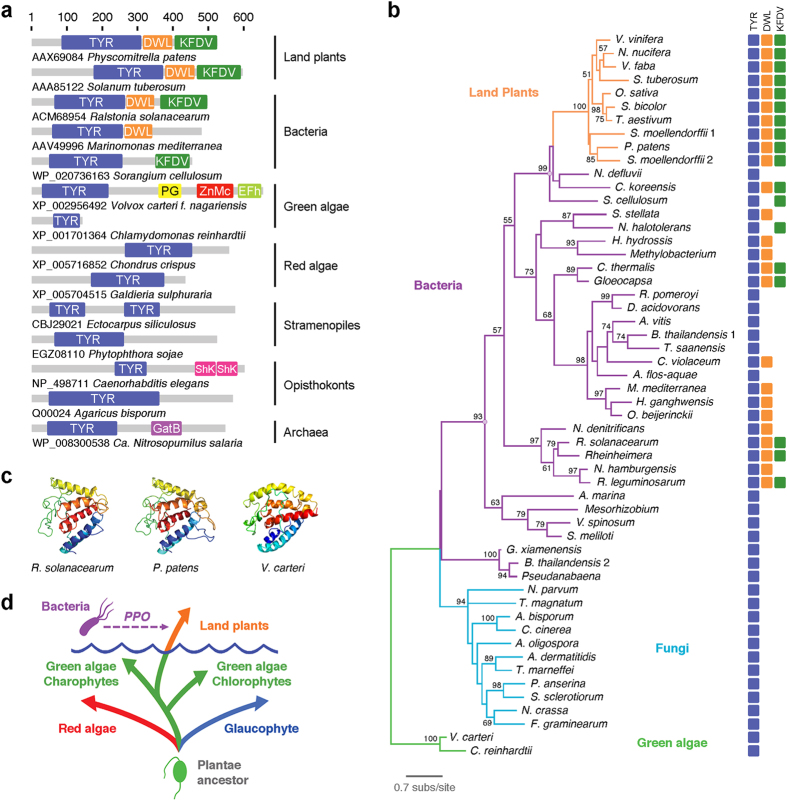
Evidence for horizontal gene transfer of *PPO* from bacteria to land plants. (**a**) Schematic protein domain architecture alignment of different representative proteins that share the tyrosinase domain (TYR; PFAM00264). The characteristic DWL (PFAM12142) and KFDV (PFAM12143) domains of land-plant PPO enzymes are also present with the same architecture in bacterial PPO proteins but appear to be absent in green and red algae, glaucophyta and other eukaryotes. Numbers on top indicate protein (amino acids) length. PG: peptidoglycan-binding domain (PFAM01471). ZnMc: zinc-dependent metalloprotease domain (CD00203). EFh: calcium-binding domain helix-turn-helix (CD00051). ShK: ShK domain-like (PFAM 01549). GatB: GatB domain (PFAM 02637). The predicted conserved domain architecture is illustrated according to CDD results. (**b**) TYR domain-derived maximum likelihood inference of phylogeny showing that land plant PPO-TYR domains form a well-supported monophyletic group with several bacteria. Taxa and branches are colour coded with fungi in light blue, bacteria in purple, land plants in orange and green algae in green. Circles indicate well-supported nodes of the plant-containing bacterial branch. Bootstrap support values above 50 are shown. Domains found in the proteins of the tree are shown as boxes on the right. (**c**) Structures of TYR domains of *R. solanacearum*, *P. patens* and *V. carteri*. Models are independently displayed based on rainbow colouring scheme (N-terminal coloured blue and C-terminal coloured red). The analysis was performed with 100% of residues modelled at >90% confidence. (**d**) Scheme of the *PPO* bacteria-to-land plants horizontal gene transfer hypothesis.

Genes coding for PPO have been found in bacteria, plants and fungi^10^,^11^, but their phylogenetic relations have not been studied in detail. At the protein level, PPOs of all three groups harbour a catalytic tyrosinase (TYR) domain (PFAM00264) towards the N-terminus, found not only in PPOs but also in several proteins of all branches of life^12^ (Fig. 1a). In plants, the C-terminal half of PPO has distinctive DWL (PFAM12142) and KFDV (PFAM12143) domains^5^. Both DWL and KFDV domains are identified as such by the Conserved Domains Database (CDD)^13^ in PPOs of various unrelated bacteria like the free-living betaproteobacterium *Chitinomonas koreensis*, the plant pathogen betaproteobacterium *Ralstonia solanacearum*, the gammaproteobacterium *Rheinheimera* sp. and the cyanobacterium *Gloeocapsa* sp. Some bacterial PPOs possess only one of these domains, while PPOs of many other bacteria as well as all fungal PPOs lack recognizable DWL and FKDV domains (Fig. 1a,b). Sequence alignments of PPO peptides from plants, bacteria and fungi show that the TYR domain can be readily aligned. The DWL and the KFDV domains, although only distantly related, can also be aligned among these proteins, indicating that the PPOs of these organisms are phylogenetically related (Supplementary Fig. S1). Notably, sequences encoding proteins with a PPO-like structure could not be retrieved from the complete genomes of the green algae *Volvox carteri*, *Chlamydomonas reinhardtii*, *Micromonas putida* and *Chlorella* sp., as well as from the genomes of red algae and other distant organisms like stramenopiles or haptophyte algae. Although some representatives of these groups have proteins containing TYR domains, DWL and KFDV domains could not be identified in any of them (Fig. 1a).

The survey described above indicates that PPO proteins are only found in land plants, fungi and some bacteria. To analyse the phylogenetic relations between these proteins, we focused on the TYR domain. A Maximum Likelihood analysis of the relationships between PPO tyrosinase (PPO-TYR) domains of land plants, bacteria and fungi reveals that land plant PPO-TYR domains form a well-supported monophyletic group with the PPO-TYR of several bacteria (Fig. 1b). Protein structure predictions of *R. solanacearum* (WP_003278615), *Physcomitrella patens* (AAX69084) and *V. carteri* (XP_002956492) further illustrate a greater structural similarity among land plant and bacterial TYR domains (Fig. 1c). The PPO-TYR domains of fungi are grouped in a separate, well-supported branch (Fig. 1b). Although green algae lack bona fide PPO proteins, we included in our phylogenetic analysis the only TYR domains that could be retrieved from the proteomes of *Chlamydomonas* and *Volvox*. As shown in Fig. 1b, the green algae TYR domains do not cluster with land plants, suggesting that land plants have not inherited PPO-TYR domains from a green algal ancestor by vertical inheritance. The patchy phylogenetic distribution of *PPO* genes as well as the phylogenetic incongruences observed for the PPO-TYR domains support the conclusion that PPO proteins are of ancient origin but have been subjected to many different HGT events during evolution. In particular, the fact that PPO-TYR domains of land plants, including the basal groups Bryophyta (*P. patens*) and Lycophyta (*Selaginella moellendorffii*), are all grouped together with bacteria and that *PPO* genes cannot be found in glaucophyte, red and green algal genomes, strongly suggest that a *PPO*-like gene was transferred from a bacterium to an early land plant ancestor circa 450–500 million years ago during the transition of plants to live on land^14^ (Fig. 1d). Additional evidence supporting the *PPO* bacteria-to-plants HGT is the observation that, compared to the average of plant genes, which typically contain 5–6 introns^15^,^16^, plant *PPOs* have substantially fewer or no introns (0–2 introns)^5^, a characteristic feature of prokaryotic horizontally acquired genes^17^.

### Recapitulation of the *PPO* prokaryote-to-eukaryote transition

**Figure 2:**
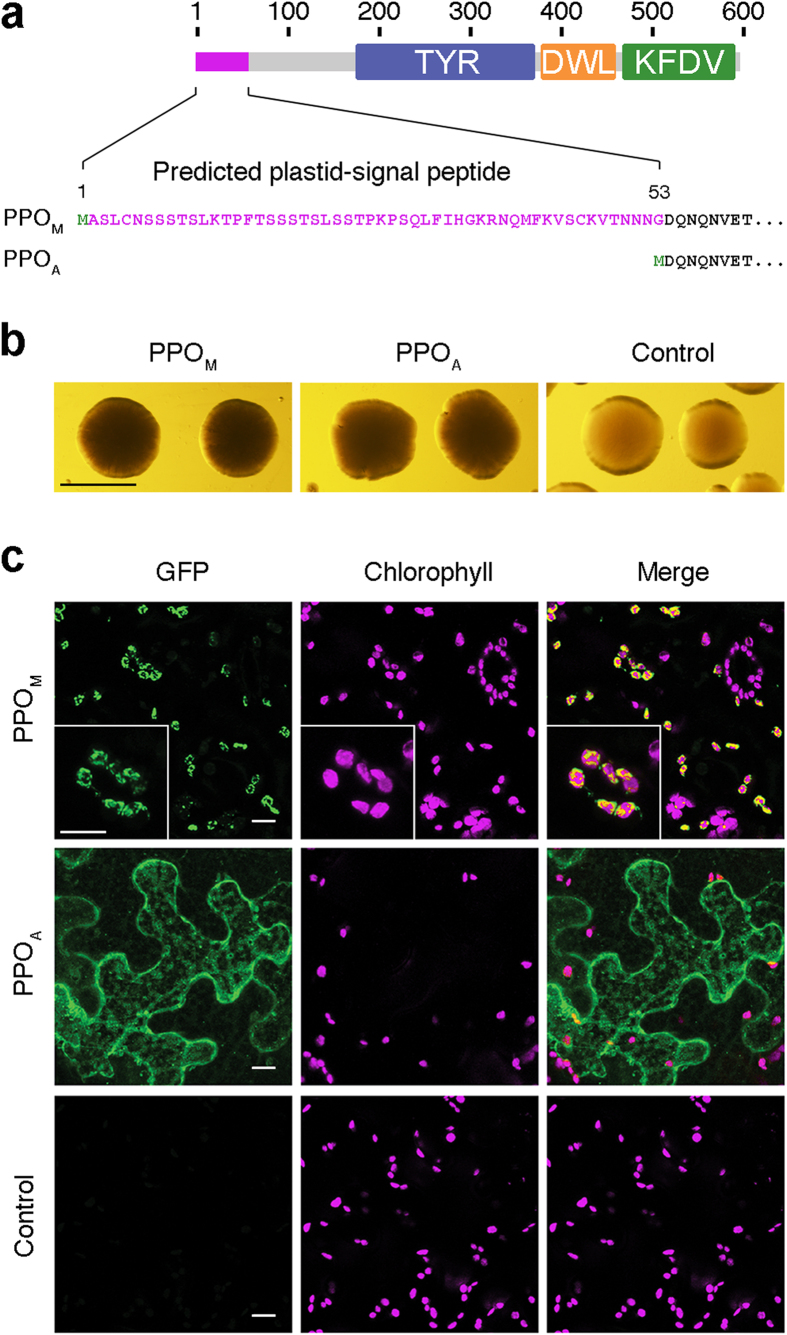
Plastidial (PPO_M_) and cytosolic (PPO_A_) PPO models. (**a**) Schematic representation of the PPO enzymes used in this study. The 53 amino acids in length predicted plastid signal peptide of PPO_M_ (depicted in magenta) was removed from protein AAA85121 (597 amino acids in length) to generate PPO_A_. Numbers on top indicate protein (amino acids) length. (**b**) Melanin production in *E. coli* cells expressing PPO_M_ and PPO_A_ compared with cells transformed with control plasmid. Images correspond to 3-day-old *E. coli* colonies grown on LB supplemented with CuSO4, IPTG and chlorogenic acid. Scale bar represents 200 μm. (**c**) Subcellular localization of PPO_M_ (plastids) and PPO_A_ (cytosol) fused to GFP in *N. benthamiana* leaves visualized by confocal microscopy. Scale bars represent 10 μm.

Having defined the bacteria-to-plants HGT of *PPO*, we set to experimentally recreate its horizontal acquisition by land plants. Our aim was to generate a model to study the effect of incorporating a nuclear gene coding for a protein of prokaryotic origin (PPO) that initially lacked information for a defined subcellular localization and eventually evolved a plastidial destination. Given that inferred ancestral proteins and proteins having evolved in distant lineages may result inactive or cause a stress derived from maladapted cell components when introduced into a distantly related host^18^,^19^, we decided to use a modified plant PPO to simulate the presumed ancestral cytosolic state of the enzyme rather than a resurrected or a bacterial protein. To ensure that the enzyme used would be active, we chose a *PPO* gene from *Solanum tuberosum* that we previously confirmed to encode an active PPO enzyme^20^. The plastid-targeting signal sequence of the *PPO* gene was identified *in silico* using ChloroP^21^. Then, two versions of this gene were cloned: one lacking the predicted plastid-targeting signal (version PPO_A_, for enzyme with Ancestral localization) and one retaining it (version PPO_M_, for enzyme with Modern localization) (Fig. 2a). The recombinant proteins were expressed in *Escherichia coli* and the activity of PPO_A_ and PPO_M_ was confirmed *in vivo* based on the ability of both proteins to induce melanin synthesis (Fig. 2b). To determine the subcellular localization of PPO_A_ and PPO_M_ in plant cells, a GFP (green fluorescent protein) tag was fused to the C-terminal end of both PPO versions and the corresponding constructs were transiently expressed in *Nicotiana benthamiana* leaves. Laser-scanning confocal microscopy analyses showed that PPO_A_ presents a cytosolic distribution pattern while PPO_M_ is detected only in plastids (chloroplasts) (Fig. 2c), as expected.

**Figure 3:**
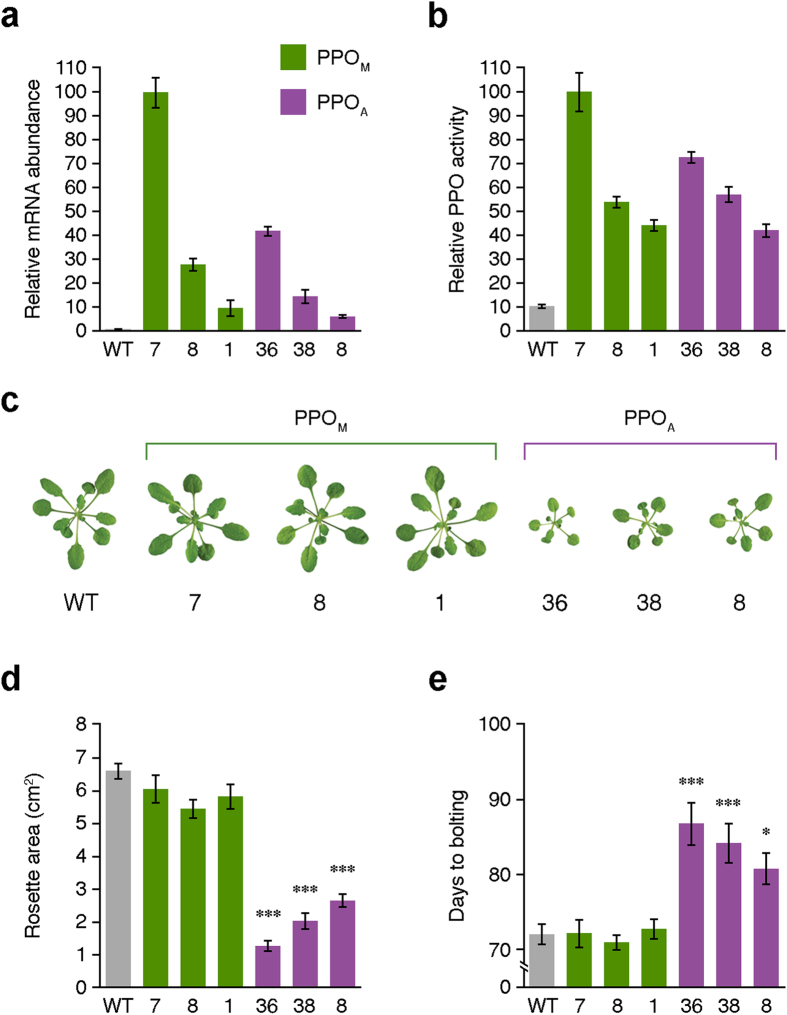
Arabidopsis plants expressing PPO_A_ have reduced growth rate and delayed bolting time. (**a**) RT-qPCR analysis of *PPO* mRNA abundance in PPO_M_ and PPO_A_ lines. Data are represented relative to PPO_M_ line 7 and correspond to mean ± SEM derived from three technical repeats of three biological replicates. Data correspond to 4-week-old plants. (**b**) PPO activity for PPO_M_ and PPO_A_ lines. Data are represented relative to PPO_M_ line 7 and correspond to mean ± SEM derived from three technical repeats of three biological replicates. Data correspond to 4-week-old plants. (**c**) Phenotype of PPO_M_ and PPO_A_ lines. Data correspond to 4-week-old plants. (**d**) Surface area of rosette leaves for PPO_M_ and PPO_A_ lines. Data correspond to mean ± SEM derived from 10 biological replicates. Asterisks represent statistically significant differences (***P = *0.001*) according to the ANOVA followed by Newman-Keuls post-hoc test. Data correspond to 4-week-old plants. (**e**) Bolting time in PPO_M_ and PPO_A_ lines. Data correspond to mean ± SEM derived from 10 biological replicates. Asterisks represent statistically significant differences (*P = *0.05*, ***P = *0.001*) according to the ANOVA followed by Newman-Keuls post-hoc test.

To experimentally study the PPO bacteria-to-plants transition (albeit in a modern context), we generated stably transformed Arabidopsis plants by *Agrobacterium*-mediated transformation with constructs for constitutive PPO_A_ or PPO_M_ expression. In agreement with the conclusion that PPO activity is not absolutely required for photosynthesis or any other primary plant function^5^,^6^,^20^,^22^, Arabidopsis does not contain any *PPO* genes^5^ and thus it has lived without PPO for several million years^23^. We therefore chose Arabidopsis as an optimal plant system to have an approximation of the physiological impact of acquiring a *PPO* gene *ab initio*. After isolating independent homozygous lines containing a single T-DNA insertion in the nuclear genome, we selected three PPO_A_ and three PPO_M_ lines with different *PPO* gene-expression (Fig. 3a) and activity (Fig. 3b) levels. Expressing the cytosolic PPO_A_ led to reduced growth rates, while expressing the plastid-localized PPO_M_ had no evident phenotypic effect on transgenic lines compared to untransformed controls (Fig. 3c–e). The expression and activity of the cytosolic version inversely correlated (r = −0.97 and r = −0.99, respectively) with the surface area of rosette leaves (Fig. 3d), supporting the conclusion that the observed deleterious phenotype in PPO_A_ lines was directly caused by the presence of such an extraplastidial PPO activity. Plants transformed with PPO_A_ were also considerably delayed in terms of bolting time (Fig. 3e), which again correlated with its expression and activity (r = 0.93 and r = 0.99, respectively).

**Figure 4:**
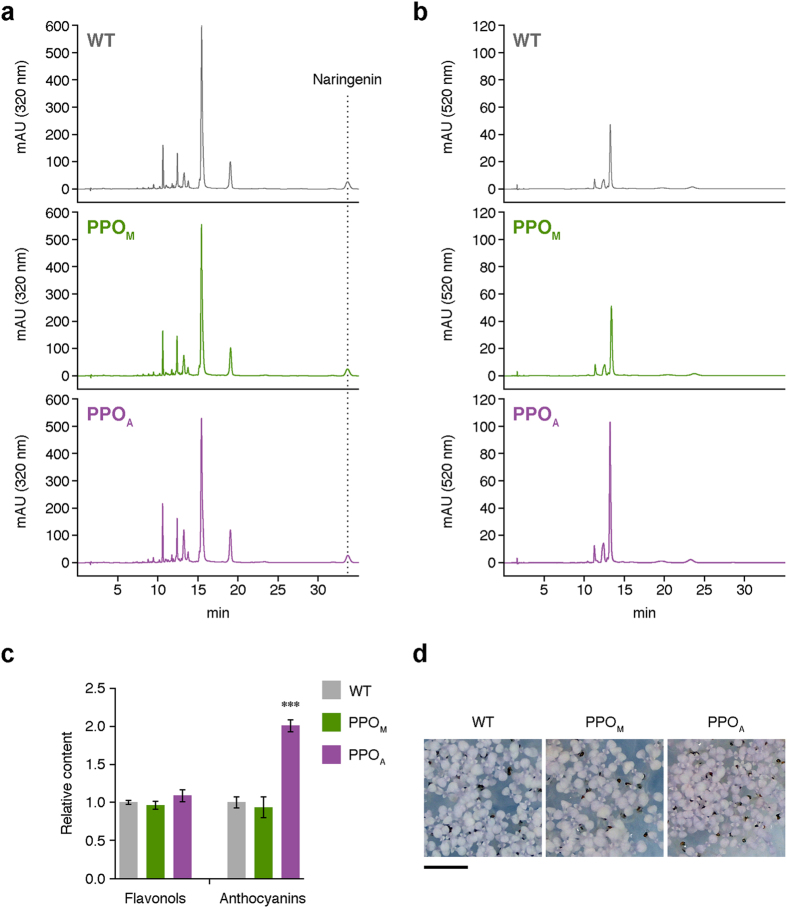
Qualitative and quantitative profiles of phenylpropanoid metabolites in transgenic and untransformed Arabidopsis seedlings. Transgenic PPO_M_ and PPO_A_ lines together with untransformed (WT) controls were grown on sucrose-supplemented medium and used for phenylpropanoid profiling as described in Materials and methods. (**a**) Representative HPLC chromatograms of flavonols. (**b**) Representative HPLC chromatograms of anthocyanins. (**c**) Total flavonol and anthocyanin levels represented relative to WT samples. Data correspond to mean ± SEM derived from three technical repeats of four biological replicates. Asterisks mark statistically significant differences (***P = *0.001*) according to the ANOVA followed by Newman-Keuls post-hoc test. (**d**) Phenotype of seedlings grown for 10 days in the presence of norflurazon. Note the stronger anthocyanin (purple) pigmentation in PPO_A_ seedlings. Scale bar represents 0.5 cm.

At the metabolic level, the qualitative profile (i.e. number of HPLC peaks) of phenylpropanoid compounds such as flavonols (Fig. 4a) and anthocyanins (Fig. 4b) was found to be very similar in PPO_A_, PPO_M_, and untransformed plants. However, the presence of an active cytosolic PPO enzyme in PPO_A_ lines had a strong quantitative impact on anthocyanin levels, which were doubled relative to PPO_M_ plants or wild-type controls (Fig. 4c), Anthocyanins are purple pigments widely used as visual markers of plant cell stress. For example, treatment with norflurazon generates albino seedlings in which the absence of photosynthetic pigments (carotenoids and chlorophylls) causes a strong photooxidative stress and a visually detected accumulation of anthocyanins that again is higher in PPO_A_ lines (Fig. 4d). Together, these results suggest that PPO enzymes can act on unidentified cytosolic substrates to eventually unchain a stress response. If such response is sustained (e.g. in lines constitutively expressing the extraplastidial PPO_A_ enzyme), a negative impact on plant growth occurs.

## Discussion

Despite the relevant contribution of HGT-acquired prokaryotic proteins to the establishment of the plastid proteome^1^,^2^, the evolutionary forces that promoted their targeting to the plastid have remained an unresolved evolutionary question. Focusing on a carefully selected candidate gene (*PPO*) for a case study, we have aimed to shed some light on this subject.

Our results strongly suggest that an early common ancestor of land plants horizontally acquired a gene coding for PPO of bacterial origin, possibly as a consequence of life in close contact with soil bacteria (Fig. 1d). An early eukaryotic origin of *PPO* is less parsimonious and would require, at least, multiple independent *PPO* losses in the glaucophyte, red algae and green algae lineages, as well as in the animal lineage. Furthermore, since the last common ancestor of the supergroup Plantae^3^ originated more than 1.2 billion years ago^24^, this scenario would also imply that the *PPO* gene was maintained for more than 700 million years only in the algal lineage leading to land plants to be then lost after the colonization of land. Interestingly, the land plant acquisition of *PPO* corresponds to the period when the phenylpropanoid biosynthetic pathway (a rich source of phenolic metabolites) emerged in land plants, also in part by the gain of horizontally acquired genes from soil bacteria or fungi^25^. These coincidental events most likely allowed unprecedented interactions between PPO and the phenylpropanoid pathway, setting the ground for evolutionary innovations that led to the development of defensive and secondary metabolic functions for PPO in land plants. For instance, keeping the PPO enzyme separated from phenylpropanoid substrates that could be converted into toxic products for invaders could have represented a novel resource-efficient defence mechanism that only becomes active when cells are attacked and components of different subcelular compartments are mixed.

It has been established that translocation of genes from the essentially prokaryotic plastid genome to the nucleus involves post-insertional genomic rearrangements rather than random integration into a genomic location conferring instant functionality^26^,^27^,^28^,^29^. On the other hand, the acquisition of plastid targeting peptides is a process that can evolve with relative ease from existing genome sequences^30^,^31^. According to these data, it is presumed that ancestral prokaryote-to-eukaryote horizontally transferred sequences typically involved an initial integration into the nuclear genome followed by the incorporation of eukaryotic transcriptional regulatory elements and, in many cases, the eventual acquisition of subcellular localization signals. Therefore, for most HGT events resulting in plastid-localized proteins, there must have been an evolutionary time when populations contained both the ancestral (i.e. non-targeted, cytosolic) version of the protein and the modern (i.e. targeted, plastid-localized) version that was ultimately retained. From a Darwinian fitness viewpoint, and independently of the defence mechanism proposed for PPO in land plants, our results allow proposing that the ancestral bacterial gene coding for a non-targeted PPO integrated in the nuclear genome of an early common ancestor of land plants, became transcriptionally active, and subsequently evolved a plastid-targeting sequence, resulting in a more beneficial variant that was hence selected. Similar to that observed in bacteria^9^, it is possible that the acquisition of a non-targeted (i.e. cytosolic) PPO by early land plants might have provided the ability to synthesize phenolic polymers or other compounds with protective effects. This putative adaptive advantage might have been turned into a disadvantage once the transition from aquatic to terrestrial environments was completed. Alternative evolutionary trajectories that also fit our results would be that, when acquired by the land-plant ancestor, the bacterial *PPO* gene integrated by chance in a genomic location simultaneously conferring both transcriptional activation and plastid targeting or that the acquisition of a plastid localization signal preceded the transcriptional activation of the gene. Under these hypotheses, eventual mutations or genomic rearrangements resulting in the loss of the plastid-localization signal would lead to a disadvantageous phenotype and the cytosolic PPO would not be retained. In either case, the finding that PPO_A_ triggers a stress response (as deduced from the accumulation of anthocyanins) that could eventually lead to reduced growth rates when compared with PPO_M_ implies that directional selection would increase the frequency of *PPO*_*M*_ relative to *PPO*_*A*_ after the horizontal acquisition of the bacterial gene, ultimately favouring the fixation of the plastidial enzyme within the population.

In stark contrast to the common assumption that newly acquired genes conferring a strongly negative fitness effect will irreversibly undergo gene degeneration or loss, we propose that the fortuitous acquisition of organelle targeting sequences can provide an alternative fate to such genes. The argument is made that a horizontally acquired prokaryotic gene coding for a delocalized protein could have a deleterious effect that is only context-specific in a eukaryotic cell where different biological processes are highly compartmentalized. The targeting of the foreign protein to an organelle such as the plastid could alleviate the deleterious effect, hence allowing the gene to endure in the recipient genome and thereafter serve as raw material for the evolution of novel functions. The plausible generality of the proposed evolutionary mechanism is supported by the well-known fact that cytosolic localization of heterologous proteins is often deleterious to plant growth when compared with plastid-localized versions^32^,^33^,^34^,^35^, probably due to interference with cytosolic metabolic pathways and the enormous capacity of the plastid to accumulate heterologous proteins.

A fundamental goal in biology is to elucidate the forces leading to evolutionary change. Previous work has revealed the unexpected broad contribution of prokaryotic proteins acquired via independent HGT events to the plastid proteome^1^,^2^, the processes leading to gene functionalization of prokaryote-like (plastid) sequences in the nucleus^26^,^27^ and the evolutionary acquisition of plastid targeting sequences^30^,^31^. By studying the likely evolutionary history followed by a horizontally transferred bacterial gene after acquisition by land plants hundreds of millions of years ago, we have experimentally shown that selective pressure against heterologous genes encoding cytosolic proteins may be an evolutionary force driving plastid proteome complexity from non-endosymbiotic gene sources. Finally, it has not escaped our notice that, as with the plastid, the inference we have postulated may also apply to certain horizontally transferred prokaryotic genes whose encoded proteins have been ultimately targeted to the mitochondrion^36^,^37^,^38^.

## Methods

### Gene sampling

Extensive BLAST^39^ searches spanning the diversity of eukaryotic and prokaryotic life were performed to identify putative eukaryotic and prokaryotic PPO homologues. Additional searches were also performed excluding land plants (embryophytes, taxid: 3193). The searches for amino acid sequences were conducted against the GenBank non-redundant protein sequences database using the BLASTp tool of the NCBI (National Center for Biotechnology Information) BLAST server. The PPO-TYR domain was also used to search the genomes of unicellular eukaryotes deposited in the Joint Genome Institute (http://genome.jgi-psf.org/) and Phytozome (www.phytozome.net). To check the robustness of the gene sampling additional searches using PSI-BLAST^39^ (5 iterations), tBLASTn and HMMER^40^ were performed.

### Phylogenetic analysis

Tyrosinase domains of PPO proteins and other TYR-containing proteins were aligned with ClustalW^41^ and edited with Jalview 2.8^42^ to minimize gaps. Information about sequences used is described in detail in Supplementary Table S1. The tree in Fig. 1b used the LG protein substitution model^43^, but JTT^44^ and Blosum62^45^ yielded essentially the same results. Bootstrapped maximum likelihood phylogenetic trees were constructed with Phyml 3.0^46^,^47^. Gamma correction was used to account for heterogeneity in evolutionary rates with four discrete classes of sites, an estimated alpha parameter and an estimated proportion of invariable sites as implemented in Phyml. A total of 100 bootstrap replicates were used to assess robustness, and tree topology was optimized by subtree pruning and regrafting (SPR). The tree was visualized and edited with TreeDyn^48^. The Fig. 1d scheme summarizing and simplifying our current understanding of the supergroup Plantae evolution was inspired by those recently published^3^,^49^,^50^.

### Protein domain architecture comparison

Genes coding for proteins with tyrosinase domains were compared by examining their corresponding protein domain architectures using CDART (Conserved Domain Architecture Retrieval Tool: http://www.ncbi.nlm.nih.gov/Structure/lexington/lexington.cgi)^51^, and the CDD (Conserved Domains Database: http://www.ncbi.nlm.nih.gov/Structure/cdd/cdd.shtml)^13^ of the NCBI.

### Structural modelling

The structural modelling was performed using the intensive modelling mode of the Protein Homology/analogy Recognition Engine (Phyre) Version 2.0 (http://www.sbg.bio.ic.ac.uk/phyre2/ html/page.cgi?id=index)^52^.

### Plasmids, plant material and growth conditions

Full-length cDNAs encoding *Solanum tuberosum PPO* (GenBank accession number: U22921) or a *PPO* version lacking localization signal were amplified from cDNA and cloned into plasmid pDONR207 using Gateway technology (Invitrogen, California, USA). The *in silico* detection of the subcellular localization signal was identified using ChloroP^21^ (http://www.cbs.dtu.dk/services/ChloroP/). Sequences were then subcloned into plasmids pGWB405^53^, pET28^54^ (modified for Gateway-compatible cloning) and pB7FWG2^55^. Constructs were confirmed by restriction mapping and DNA sequence analysis. Information about primers used is described in detail in Supplementary Table S2. Constructs in pGWB405 were used for transient expression in *Nicotiana benthamiana* leaves^56^. Constructs in pET28 were used for protein expression in *Escherichia coli*. Constructs in pB7FWG2 were used for stably *Agrobacterium tumefaciens*-mediated transformation of *Arabidopsis thaliana* plants (ecotype Col-0)^57^. Homozygous Arabidopsis lines containing a single T-DNA insertion were selected based on the segregation of the phosphinothricin (PPT) resistance marker. Initial segregation analysis was performed by seeding plants on sterile Murashige and Skoog (MS) medium plates supplemented with 20 μg/mL PPT (Duchefa, Haarlem, Netherlands). A total of 25 PPO_M_ and 29 PPO_A_ homozygous lines were isolated; from which three independent lines of each PPO version were selected for further characterization. Arabidopsis plants were grown on soil in a climate controlled growth chamber (22 °C, 65–70% RH, and 60 μmol m^−2^ s^−1^ photosynthetically active radiation) under 8 h of light/16 h of dark photoperiod for 4 weeks. For phenylpropanoid profiling, Arabidopsis seeds were surface-sterilized and sown on Petri plates with sterile MS medium containing 1% agar and 30 g/L sucrose. When indicated, plates were supplemented with 5 μM norflurazon. After stratification for 3 days at 4 °C in the dark, the plates were incubated in growth chambers for 20 days at 22 °C under continuous light (130 μmol m^−2^ s^−1^ photosynthetically active radiation).

### Expression of recombinant PPO_A_ and PPO_M_ in *E. coli* cells

Following transformation of competent *E. coli* cells strain Rosetta 2 (DE3) (Novagen, Merck KGaA, Darmstadt, Germany) with purified plasmids pET28-PPO_A_ and pET28-PPO_M_, the activity of PPO_A_ and PPO_M_ was confirmed by growing the transformed *E. coli* cells for three days at 28 °C on LB agar plates supplemented with 34 μg/mL chloramphenicol, 25 μg/mL kanamycin, 0.5 mM IPTG, 40 μg/ml CuSO_4_, and 600 μg/ml chlorogenic acid, similarly to methods previously described^58^ to detect melanin formation. PPO_A_ and PPO_M_ colonies were dark because the cells were able to produce melanin-like polymers.

### Confocal laser-scanning microscopy

Subcellular localization of GFP fluorescence and chlorophyll fluorescence was determined with an Olympus FV 1000 confocal laser-scanning microscope (Olympus, Tokyo, Japan) using an argon laser for excitation (at 488 nm) and a 500–510 nm filter for detection of GFP fluorescence and a 610–700 nm filter for detection of chlorophyll fluorescence.

### Measurement of rosette leaves surface area

The surface area of rosette leaves was measured using Photoshop (Adobe, California, USA) and GraphPad Prism 5.0a (GraphPad Software, California, USA) by analysing digital images (acquired from above) of 4-week-old plants and comparing them with size standard series ranging from 4 to 16 cm^2^.

### Transcripts analysis

Leaf samples from 4-week-old plants were harvested and total RNA was extracted using the Maxwell 16 LEV simplyRNA Tissue Kit (Promega, Wisconsin, USA) according to the manufacturer’s instructions. Purified RNAs were quantified by spectroscopy using a NanoDrop apparatus (Thermo Scientific, Massachusetts, USA) and RNA integrity was evaluated by agarose gel electrophoresis. The First Strand cDNA Synthesis Kit (Roche, Basel, Switzerland) was used to generate cDNA according to the manufacturer’s instructions, using 50 pmol of an anchored poly(dT) primer [d(T_18_V)] and 2 μg of total RNA. The relative mRNA abundance was evaluated via quantitative reverse transcription PCR (RT-qPCR) in a total reaction volume of 20 μl using LightCycler 480 SYBR Green I Master (Roche, Basel, Switzerland) on a LightCycler 480 Real-Time PCR System (Roche, Basel, Switzerland) with 0.3 μM of each specific sense and anti-sense primers. Three independent biological replicates of each sample and three technical replicates of each biological replicate were performed and the mean values were considered for further calculations. The normalized expression of *PPO* was calculated as described^59^ using *UBC* (Arabidopsis ubiquitin-conjugating enzyme gene At5g25760, GenBank accession number: DQ027035) as the endogenous reference gene. Information about primers used is described in detail in Supplementary Table S2.

### PPO activity

Protein extracts were obtained from rosette leaves homogenized at a ratio of 50 mg of fresh weight to 1000 μl of 20 mM phosphate buffer (pH 6). The homogenate was centrifuged at 16.000 × *g* and 4 °C for 5 min, and the supernatant was used for protein assessment of PPO activity. PPO activity assays were performed in 20 mM phosphate buffer (pH 6) containing 10 mM L-DOPA. The reaction was measured with a SpectraMax M3 spectrophotometer (Molecular Devices, California, USA) by the change in absorbance at 475 nm and 25 °C.

### Phenylpropanoid profiling

Phenylpropanoids (specifically, flavonoids) were purified and analysed as described previously^60^ with minor modifications. In brief, lyophilized Arabidopsis seedling were homogenized at a ratio of 1 mg of dry weight to 33 μL of methanol:acetate:H_2_O (9:1:10) extraction solvent containing 0.1 mg/mL of naringenin as an internal standard for quantification. Homogenates were incubated in the dark at 4 °C for 30 min with agitation (1000 rpm) and then centrifuged at 14,000 × *g* and 4 °C for 5 min. Supernatants were recovered, filtered and analysed by high-performance liquid chromatography (HPLC) using an Agilent 1200 series HPLC equipment (Agilent Technologies, California, USA) with a XSelect CSH C18 (3.5 μm, 4.6 × 100 mm) column (Waters, Massachusetts, USA). Flavonols and anthocyanins were determined at 320 and 520 nm, respectively.

### Data analysis

ANOVA followed by Newman-Keuls multiple comparison post-hoc tests were used to determine statistical significance for multiple groups. Pearson correlation coefficients (*r* values) were calculated using the means of the surface area of rosette leaves, *PPO* mRNA abundance and PPO activity. Statistical analysis was performed using GraphPad Prism 5.0a (GraphPad Software, California, USA).

## Additional Information

**How to cite this article**: Llorente, B. *et al.* Selective pressure against horizontally acquired prokaryotic genes as a driving force of plastid evolution. *Sci. Rep.* **6**, 19036; doi: 10.1038/srep19036 (2016).

## Supplementary Material

Supplementary Information
