# Supplementary material for: Selective pressure against horizontally acquired prokaryotic genes as a driving force of plastid evolution
Source: Sci Rep. 2016 Jan 11;6:19036. doi: 10.1038/srep19036 (PMC4707469; doi:10.1038/srep19036)
Supplement: Supplementary Information [file srep19036-s1.pdf]

Supplementary Information

Selective pressure against horizontally acquired prokaryotic genes as a driving force of plastid evolution

Briardo Llorente[1,2,*], Flavio S. J. de Souza[2,+], Gabriela Soto[2,+], Cristian Meyer[2], Guillermo D. Alonso[2,3], Mirtha M. Flawiá[2,3], Fernando Bravo-Almonacid[2,4], Nicolás D. Ayub[5,6] and Manuel Rodríguez-Concepción[1,*]

[1] Centre for Research in Agricultural Genomics (CRAG) CSIC-IRTA-UAB-UB, 08193 Barcelona, Spain.

[2] Instituto de Investigaciones en Ingeniería Genética y Biología Molecular Dr. Héctor Torres, Consejo Nacional de Investigaciones Científicas y Técnicas (INGEBI-CONICET), C1428ADN Buenos Aires, Argentina.

[3] Departamento de Fisiología, Biología Molecular y Celular, Facultad de Ciencias Exactas y Naturales, Universidad de Buenos Aires, C1428EGA Buenos Aires, Argentina.

[4] Departamento de Ciencia y Tecnología. Universidad Nacional de Quilmes, B1876BXD Bernal, Argentina.

[5] Instituto de Genética Ewald A. Favret, Centro de Investigación en Ciencias Veterinarias y Agronómicas, Instituto Nacional de Tecnología Agropecuaria (CICVyA-INTA), B1712WAA Castelar, Argentina.

[6] Consejo Nacional de Investigaciones Científicas y Técnicas (CONICET), C1033AAJ Buenos Aires, Argentina.

* Corresponding authors: briardo.llorente@cragenomica.es or manuel.rodriguez@cragenomica.es

+ These authors contributed equally to this work.

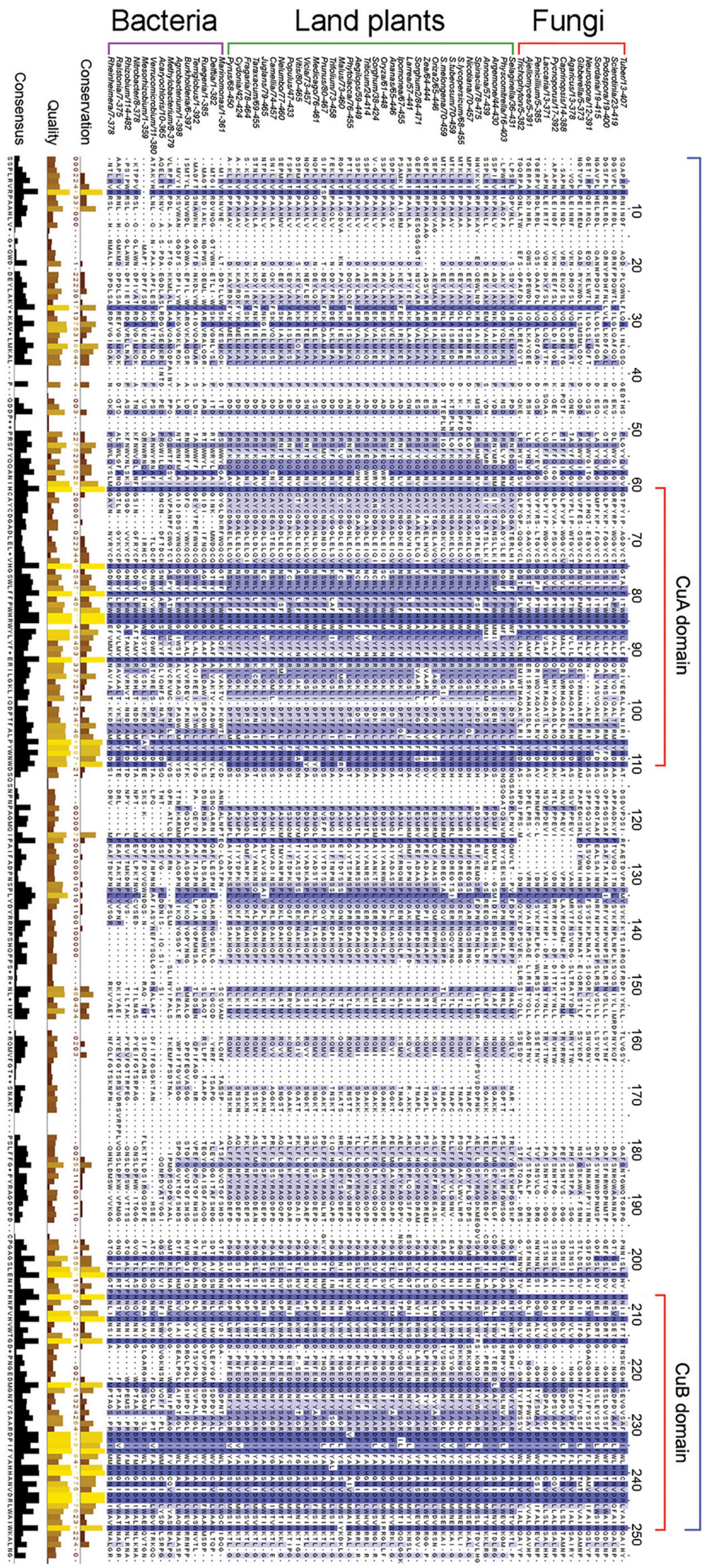

Figure S1 continues in next page

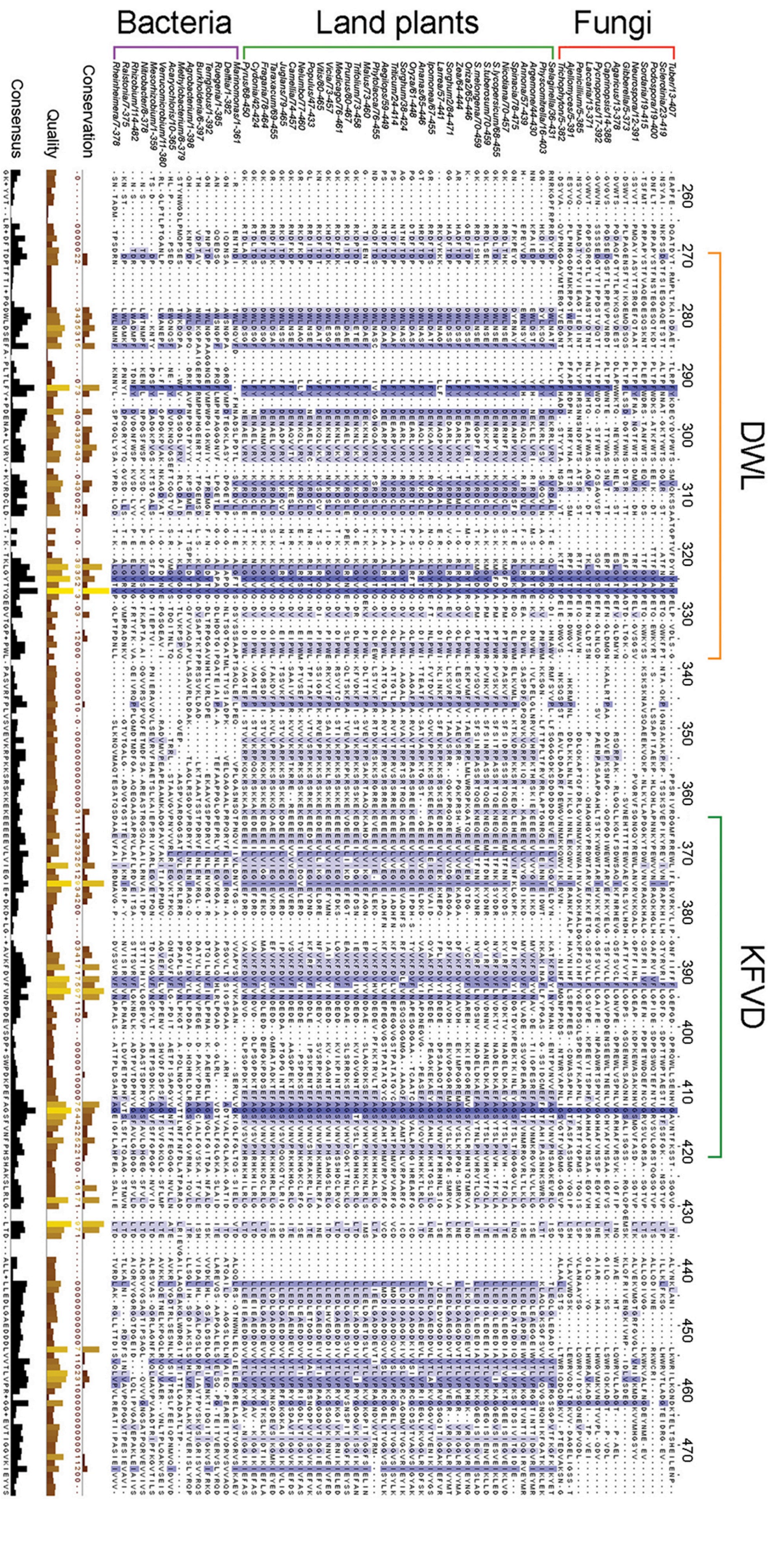

Figure S1 l Sequence alignments of PPO domains from bacteria, land plants and fungi. Alignments include the TYR (PFAM00264), DWL (PFAM12142) and KFDV (PFAM12143) domains of PPO. The highly conserved copper-binding motifs A (CuA) and B (CuB) of the TYR domain are also indicated.

Table S1. GenBank accession IDs for protein sequences used in Figure 1b.

Organism	Accession number	Phylum
Acaryochloris marina	WP_012167220	Cyanobacteria
Agaricus bisporum	Q00024	Basidiomycota
Agrobacterium vitis	WP_015916152	Proteobacteria
Ajellomyces dermatitidis ER-3	EEQ88565	Ascomycota
Aphanizomenon flos-aquae	WP_027401937	Cyanobacteria
Arthrobotrys oligospora ATCC 24927	EGX43307	Ascomycota
Burkholderia thailandensis 1	WP_015604107	Proteobacteria
Burkholderia thailandensis 2	WP_011400974	Proteobacteria
Candidatus Nitrospira defluvii	WP_013249721	Nitrospirae
Chitinimonas koreensis	WP_028446804	Proteobacteria
Chlamydomonas reinhardtii	XP_001701364	Chlorophyta
Chromobacterium violaceum ATCC 12472	AAQ60926	Proteobacteria
Chroococcidiopsis thermalis	WP_015153379	Cyanobacteria
Coprinopsis cinerea okayama7#130	XP_002911630	Basidiomycota
Delftia acidovorans	WP_016447903	Proteobacteria
Gallaecimonas xiamenensis	WP_008484930	Proteobacteria
Fusarium graminearum	CEF86307	Ascomycota
Gloeocapsa sp. PCC 7428	WP_015187306	Cyanobacteria
Hahella ganghwensis	WP_020405703	Proteobacteria
Haliscomenobacter hydrossis	WP_013766505	Bacteroidetes
Marinomonas mediterranea	AAV49996	Proteobacteria
Mesorhizobium	WP_013530378	Proteobacteria
Methylobacterium sp. 4-46	WP_012335039	Proteobacteria
Nelumbo nucifera	ADC92563	Streptophyta
Neofusicoccum parvum UCRNP2	XP_007587508	Ascomycota
Neurospora crassa OR74A	XP_964823	Ascomycota
Nisaea denitrificans	WP_028466395	Proteobacteria
Nitrobacter hamburgensis	WP_011511370	Proteobacteria
Nocardiopsis halotolerans	WP_040684307	Actinobacteria
Oceanospirillum beijerinckii	WP_028299139	Proteobacteria
Oryza sativa	ACS15320	Streptophyta
Physcomitrella patens	AAX69084	Streptophyta
Podospora anserina S mat+	XP_001912783	Ascomycota
Pseudanabaena sp. PCC 6802	WP_019501743	Cyanobacteria
Ralstonia solanacearum	ACM68954	Proteobacteria
Rheinheimera sp. A13L	WP_008899933	Proteobacteria
Rhizobium etli CFN 42	AAM54973	Proteobacteria
Ruegeria pomeroyi	WP_011047142	Proteobacteria
Sagittula stellata	WP_005858917	Proteobacteria
Sclerotinia sclerotiorum 1980 UF-70	XP_001584564	Ascomycota
Selaginella moellendorffii 1	XP_002969567	Streptophyta
Selaginella moellendorffii 2	XP_002990879	Streptophyta
Sinorhizobium meliloti	WP_017267834	Proteobacteria
Solanum tuberosum	AAA85122	Streptophyta
Sorangium cellulosum	WP_020736163	Proteobacteria
Sorghum bicolor	XP_002446945	Streptophyta
Talaromyces marneffei ATCC 18224	XP_002149601	Ascomycota
Terriglobus saanensis	WP_013568839	Acidobacteria
Triticum aestivum	AAS00454	Streptophyta
Tuber magnatum	CAT00093	Ascomycota
Verrucomicrobium spinosum	WP_009958178	Verrucomicrobia
Vicia faba	Q06215	Streptophyta
Vitis vinifera	NP_001268045	Streptophyta
Volvox carteri	XP_002956492	Chlorophyta

Table S2. Primers used in this study.

Primer name	Sequence
$StPPO_M$ F (C)	GGGGACAAGTTTGTACAAAAAAGCAGGCTATGGCAAGCTTGTGCAATAGTAGTAG
$StPPO_A$ F (C)	GGGGACAAGTTTGTACAAAAAAGCAGGCTATGGACCAAAACCAAAACGTTGAAAC
StPPO R (C)	GGGGACCACTTTGTACAAGAAAGCTGGGTTTAACAATCTGCAAGACTGATCGTCG
StPPO C-tag R (C)	GGGGACCACTTTGTACAAGAAAGCTGGGTTACAATCTGCAAGACTGATCGTCG
$StPPO_M$ N-tag F (C)	GGGGACAAGTTTGTACAAAAAAGCAGGCTTCATGGCAAGCTTGTGCAATAGTAGTAG
$StPPO_A$ N-tag F (C)	GGGGACAAGTTTGTACAAAAAAGCAGGCTTCATGGACCAAAACCAAAACGTTGAAAC
StPPO qPCR F (Q)	TGAATGCGAATGAGCTTGAC
StPPO qPCR R (Q)	CAGCTGGAAATCAACAGTCG
AtUBC qPCR F (Q)	TCAAATGGACCGCTCTTATC
AtUBC qPCR R (Q)	CACAGACTGAAGCGTCCAAG

The names are labelled with (C) or (Q) to indicate whether they were used for cloning or qPCR, respectively.
